# Investigation of Kenaf Paper in the Presence of PVA for Transformers Application

**DOI:** 10.3390/ma13215002

**Published:** 2020-11-06

**Authors:** Muhammad Umair, Norhafiz Azis, Rasmina Halis, Jasronita Jasni

**Affiliations:** 1 Advanced Lightning, Power and Energy Research Centre (ALPER), Department of Electrical and Electronic Engineering, Universiti Putra Malaysia, Serdang 43400, Selangor, Malaysia; jas@upm.edu.my; 2 Institute of Advanced Technology (ITMA), Universiti Putra Malaysia, Serdang 43400, Selangor, Malaysia; 3 Department of Natural Resource Industry, Faculty of Forestry and Environment, Universiti Putra Malaysia, Serdang 43400, Selangor, Malaysia; rasmina@upm.edu.my

**Keywords:** kenaf fiber, insulation paper, polyvinyl alcohol, physio-mechanical, AC breakdown test, beating

## Abstract

This paper presents an investigation on the physio-mechanical properties and AC breakdown voltage of the Kenaf paper in the presence of Polyvinyl Alcohol (PVA) for transformers application. Kenaf bast fibers were used in order to produce the paper through the soda pulping process. The pulps were subjected to beating up to 12,000 revolutions, whereby the PVA was added to the pulps at a different weight percentage concentration up to 12%. Morphological study was carried out on the Kenaf paper based on Scanning Electron Microscopy (SEM). The apparent density, Tensile Index (TI), Burst Index (BI), Tear Index (TeI), and AC breakdown voltage of the Kenaf paper were measured. It is found that the TI and BI of Kenaf paper can be slightly improved through the introduction of PVA. On other hand, the TeI of the Kenaf paper decreases with the increment of the PVA. The AC breakdown voltage of the Kenaf paper slightly increases with the increment of PVA weight percentage concentration.

## 1. Introduction

Wood based insulation papers have been used for decades as insulating materials in transformers, owing to its good physio-mechanical and electrical properties [1,2]. Even though it is sustainable, it can be subjected to shortages under extreme deforestation [1,2,3]. Recently, significant efforts have been carried out in order to explore the viability of non-wood fibers application as electrical insulation. Currently, non-wood fibers have been used in the pulp and paper industries. Although the non-wood based papers are inferior to wood based papers, several treatments can improve its characteristics [3,4]. Several non-wood plants have been used for papermaking, such as bamboo, Kenaf, baggase, jute, and cotton [4].

Kraft and soda processes are among the approaches used for pulping the non-wood plants. The soda process is mostly preferred for the pulping non-wood fibers, since it only requires Sodium Hydroxide (NaOH) and the production time is shorter than Kraft process [5]. Soda process could produce high yields, without affecting the overall quality of the end products [5,6]. This process results in pulps with high insoluble carbohydrates. It is also found that the strength and lignin contents of pulps that are made from soda process are almost identical with Kraft process [5,6,7].

Cellulose is known as the principal component for both non-wood and wood fibers [8]. The lengths of wood fibers are slightly longer than non-wood fibers [8,9]. The chemical compositions and morphological characteristics, such as fibers width and length, determine the paper quality. The average fiber length of non-wood plants is between 0.6 mm and 30 mm [10]. Lumen size and thickness of the cell wall affect the rigidity and strength of non-wood fiber papers [4,10]. Fibers with large lumens and thin walls could be flattened once subjected to pulping. This process increases the contacts among the fibers and lead the increment of the strengths. Nowadays, the papermaking that is based on the non-wood fibers has attracted demands in wide range of applications [9]. Previously, the use of non-wood fibers is mainly for writing papers, but, in recent years, its applications have expanded in other areas, such as tissue, printing papers, and corrugated boards [7,8,9].

Non-wood fibers such as cotton, manila, hemp and flax have also been used for insulation applications, such as cables and telephones [11,12,13,14]. These fibers have good strength, low cost, good elasticity, and flexibility properties. In addition, it can comply with the size requirements and manufacturing processes [12]. Cotton fibers have been used in transformer insulation, but it has limited thermal capacity and high moisture absorption [14]. Flax fibers have been used as insulation in the capacitor [11,12,13,14]. The combination of wood fibers and manila hemp fibers has also been used for telephone insulation [11,12,13,14]. Currently, there are limited studies that have been carried out in order to examine the suitability of Kenaf fibers as an alternative electrical insulation for transformers.

Kenaf is one of the non-wood plants that have been used in twine, coarse cloth, rope, animal bedding, packaging, and paper industries. Kenaf fibers have also been utilized for writing papers and newsprints due to its high strength characteristics of fibers [15,16,17,18]. Kenaf plants consist of core and bast fibers that are short and long, respectively [16,18]. Bast fibers are longer than core fibers ranging from 1.15 mm to 4.03 mm [18]. Long fibers are one of the main requirements for the transformer’s insulation since it needs to withstand the possible mechanical movements and heat while in-service [8,11,12,13,14]. The width of Kenaf fibers is between 13.8 µm and 30 µm and it is dependent upon the age, position, and species [10,18].

Non-wood paper production is basically a two-step process whereby the fibrous raw materials are first processed into pulps [19]. Pulping is carried out in order to separate fibers from dust and other unwanted chemicals [19]. The beating and refining of fibers are carried out to form thin fiber slurry that is suspended in solution [20]. The fiber network is built on a thin screen and it is pressed to maximize density [21]. The fiber network is further dried to extract residual moisture [22]. The beating process increases the bonding between fibers by fibrillation, thus increases the fiber surface contact area [20,21,22,23,24,25].

The performance of the paper can be improved through the introduction of chemical additives. There are two types of additives known as functional and process chemicals. The functional chemicals increase the paper properties directly while the process chemicals influence operations on or near paper machines [25,26,27]. Process chemicals are retention aids, biocides, dispersants, and defoamers [26,27,28,29]. Functional chemicals, such as fillers, sizing agents, dyes, optical brighteners, and wet/dry additives can be used in order to enhance or modify the specific characteristics of the papers [28]. It can be introduced internally or layered on the surface of the sheet [28]. Hydrogen bonds and friction forces between fibers are the main forces that determine the strength of fiber interactions in the papers whereby it can be improved by the functional chemicals [29,30,31,32].

Polyvinyl Alcohol (PVA) is one of the water-soluble synthetic polymers that has been used as functional chemical additives in papermaking [29,30]. To remove the acetate groups, PVA is subjected to partial or complete polyvinyl acetate hydrolysis, while other vinyl polymers are formed by the polymerization of its individual monomers [29,30,31,32]. PVA has been widely used in several applications, such as textiles, paper, fiber, ceramics, and wood [29,31]. PVA could enhance the paper resistance to oils and fats [29]. It has also been used with biopolymers and other hydrophilic polymers with the purpose of increasing paper’s mechanical properties due to its structural compatibility and hydrophilic characteristics [29,30,31,32].

In this paper, Kenaf paper for electrical insulation purpose has been developed and fabricated. The Kenaf pulps are prepared and subjected to beating up to 12,000 revolutions. PVA has been introduced in the Kenaf pulps in order to further improve its physio-mechanical properties and AC breakdown voltage. Tensile Index (TI), Burst Index (BI), Tear Index (TeI), thickness, apparent density, and AC breakdown voltage of the Kenaf paper are measured and analyzed. The main contribution of the study is regarding the development of the Kenaf paper with PVA as the enhancement material for application in transformers. The findings of the results can be the basis for further improvement of Kenaf paper for purpose of electrical insulation.

## 2. Preparation of Kenaf Paper

**Figure 1:**
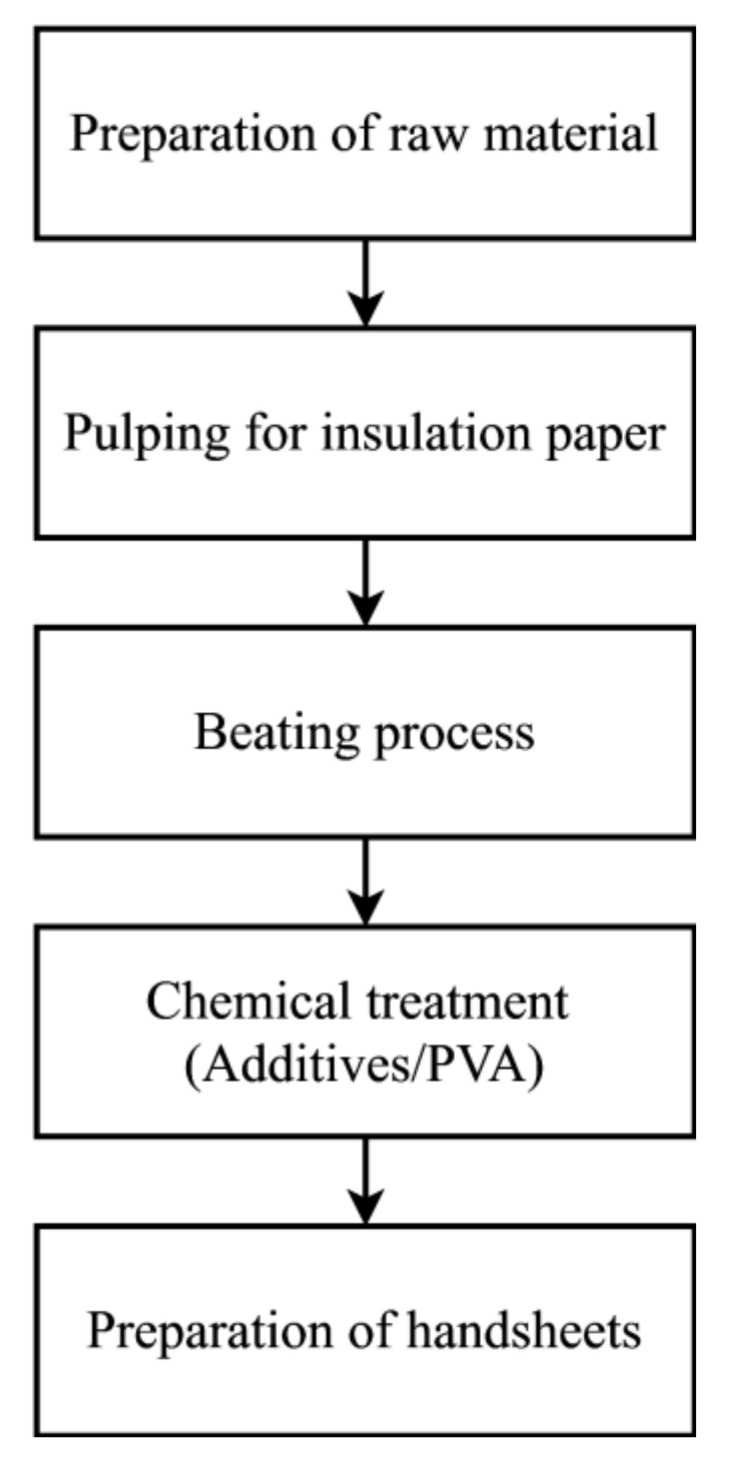
The process to produce Kenaf insulation paper.

Figure 1 illustrates the process of producing the Kenaf insulation papers. The raw material for the pulping was Kenaf bast fibers and it was obtained locally. In order to prepare Kenaf fibers for pulping, fiber threads were screened and thoroughly cleaned from dust and grime [33]. The threads were cut into 10 cm of length to make a total of 1 kg of fibers based on the Oven Dry (OD) method. Next, the pulping for Kenaf bast fibers was carried out based on the soda pulping method. Beating process was performed on the Kenaf bast fiber pulps. The speed of the beating was set at 3000 revolutions step and it was increased up to 12,000 revolutions [33]. The chemical treatment was performed by wet-end, whereby PVA was added with weight percentage concentration ranging from 3% to 12%. Wet-end is a phase of papermaking process where the pulp is in slurry form (mixture of fiber and water). Finally, the handsheets were prepared for the physio-mechanical and electrical tests.

### 2.1. Pulping Process

The Kenaf fibers were treated by soda pulping process through a rotary digester. For the pulping parameters, the concentration of NaOH was set to 14% with liquor to wood ratio of 7:1, respectively. The initial temperature of the pulping was set at 35 °C with a pressure of 140 psi. It took 90 min. to reach 170 °C whereby it was maintained for 30 min. [33].

**Figure 2:**
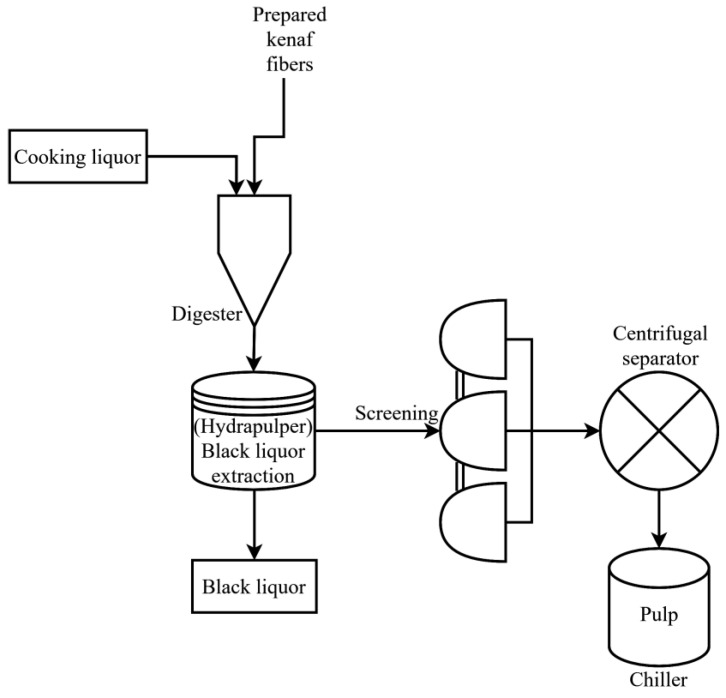
Pulping process of Kenaf fibers.

Figure 2 shows the soda pulping process of the Kenaf fibers for insulation papers. The Kenaf fibers, together with NaOH and distilled water, were fed into the digester for cooking based on the pulping parameters. After the process black liquor has been produced as by-products whereby the pulp was extracted and further cleaned with water. Black liquor mainly consists of lignin, hemicellulose and other extractives from the fibers after pulping, leaving behind mostly cellulose. After the separation of black liquor from fibers, the pulps still contained coarse fibers, dust, bark, and digester brick, which were removed by the screening process. A centrifugal separator was used to remove the excess water from the pulp. Next, the pulp was stored in a chiller at 6 °C. The percentage of yield for the pulping was 59.17%, and it was determined based on Equation (1).

(1) $$Yield\;\left(\%\right)=\frac{OD\;weight\;of\;pulp\;\left(g\right)}{OD\;weight\;of\;raw\;material\;\left(g\right)}\times 100$$

### 2.2. Freeness of Pulp

**Figure 3:**
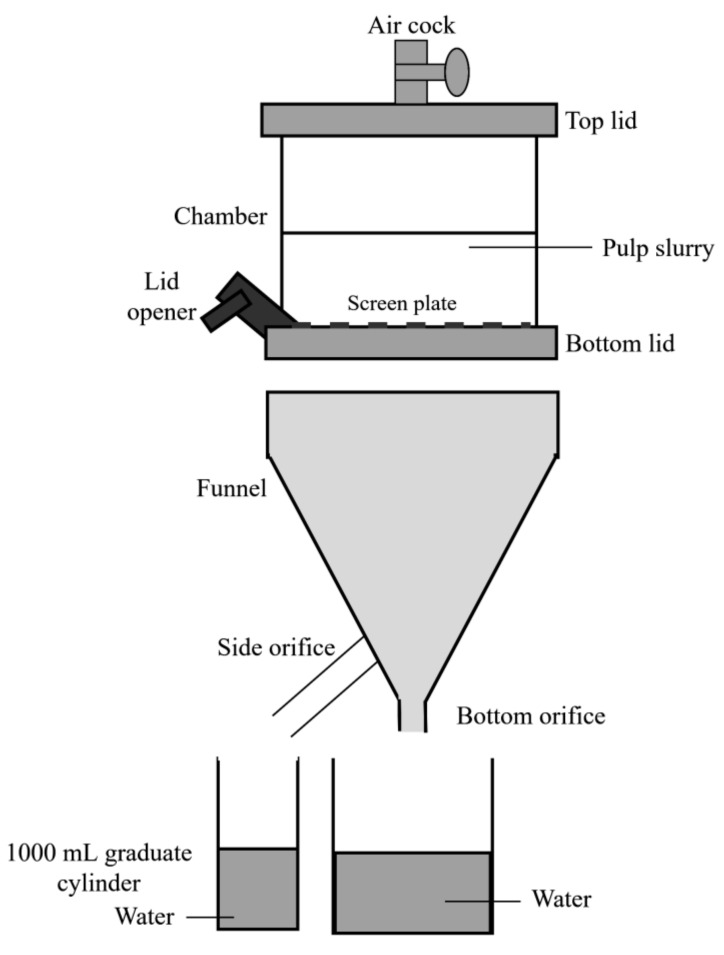
Pulp freeness measurement.

The pulp freeness was measured in order to determine the level of drainage for a condensed pulp suspension as well as to examine the surface conditions and swelling of the Kenaf fibers. The pulp’s freeness was measured according to TAPPI T227 [34]. A freeness tester, which consisted of a drainage chamber and measuring funnel was used to measure the pulp freeness, as shown in Figure 3. The tester was first thoroughly washed with distilled water. Drainage chamber was placed on the upper support bracket with its lower lid closed and both upper lid and air-cock opened. The graduate cylinder was placed in positions to receive the discharged from the side orifice and a container to collect the discharge from the bottom orifice. Next, 1 L of suspended pulp was taken from a stock divider and then poured into the chamber. The top of the lid and air cock were closed and, subsequently, the bottom lid was opened, which discharged the pulp suspension through the side orifice. The volume that was discharged from the side orifice was recorded in millimeter, which represented the freeness of pulp.

### 2.3. Classification of Kenaf Fibers

**Figure 4:**
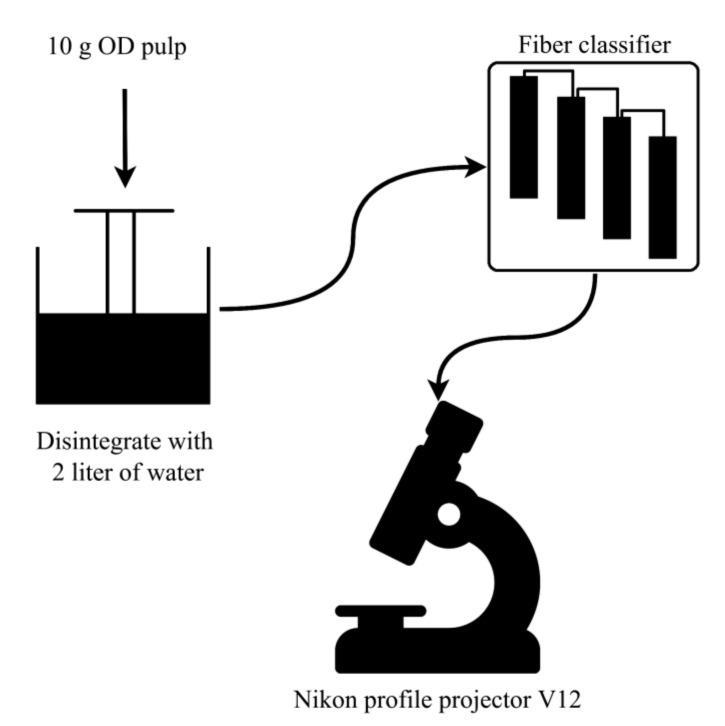
Classification of Kenaf fibers.

Baur MC Nett fiber classifier was used to determine the characteristics of Kenaf fibers while using Kenaf pulp as per TAPPI T233 [35]. In total, 10 g OD pulp was added in 2 L of water for 4 h. Next, a mixer was used to disintegrate the solution in order to produce homogenous pulp suspension, as shown in Figure 4. The pulp suspension was subjected to a fiber classifier fitted with 4 meshes that consisted of filter papers. The long fibers in the pulp suspension maintained on the first mesh, while the short fibers remained on the final mesh. The lengths of fibers were measured by a Nikon profile projector V12 after drying the filter paper for 24 h at room temperature.

### 2.4. Beating Process

**Figure 5:**
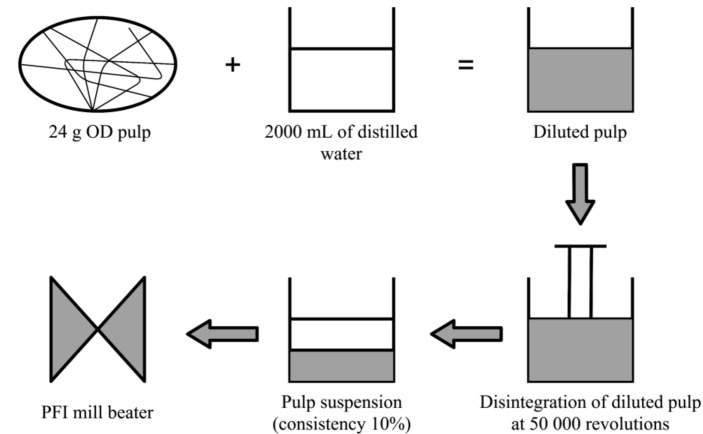
Beating process of the Kenaf pulps.

The pulps were subjected to the beating procedures by Noram PFI mill in accordance with TAPPI T248 [36]. 24 g OD pulp was diluted with 2 L of distilled water and it was disintegrated at 50,000 revolutions. First, the pulp consistency, C, was set to 10% based on Equation (2) before the beating was performed [36]. The beating interval was set at 3000 revolutions and it was increased to 12,000 revolutions, as shown in Figure 5.

(2) $$C=\frac{ODP}{WP}\times 100$$

where,

*C* = consistency of pulp (%)
*ODP* = oven dry weight of pulp (g)
*WP* = weight of pulp (g)

### 2.5. Chemical Treatment

**Figure 6:**
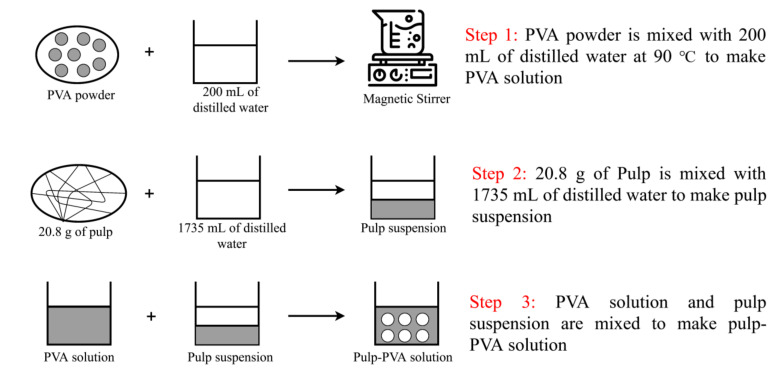
Polyvinyl alcohol (PVA) treatment on the Kenaf pulps.

The PVA powder was added to the pulp at weight percentage concentrations between 3% and 12%. The weight percentage concentration of PVA was calculated based on Equation (3), according to the OD weight of pulp. It was mixed with 200 mL of distilled water at 90 °C while using magnetic stirrer for 2 h, in order to produce homogenous PVA solution as shown in Figure 6. The PVA solution was then cooled at room temperature. In order produce the pulp suspension, 20.8 g of pulp was diluted with 1735 mL of distilled water. A mixer was used to disintegrate PVA solution and pulp suspension at 50,000 revolutions.

(3) $$\%\mathrm{PVA}=\frac{Weight\;of\;PVA\;\left(g\right)}{Total\;weight\;of\;pulp\;\left(g\right)}\times 100$$

### 2.6. Preparation of Handsheets

**Figure 7:**
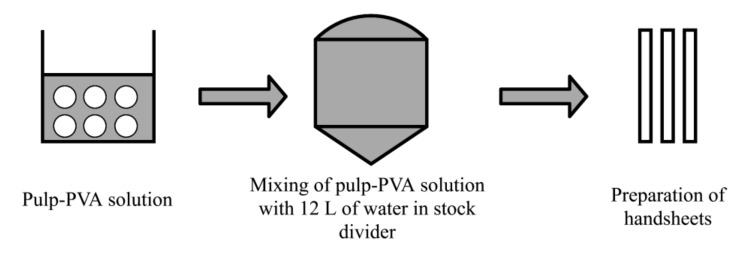
Papermaking process.

Figure 7 shows the process for producing handsheets. Handsheets with the grammage of 52 g/m^2^ were produced according to TAPPI T205 [37]. The pulp-PVA solution was poured into the stock divider and then mixed with 12 L of distilled water. In total, 867 mL of pulp suspension was obtained from the stock divider in order to produce the handsheets on paper machine.

## 3. Experimental Setup

### 3.1. Pre-Processing of Oil and Paper

**Figure 8:**
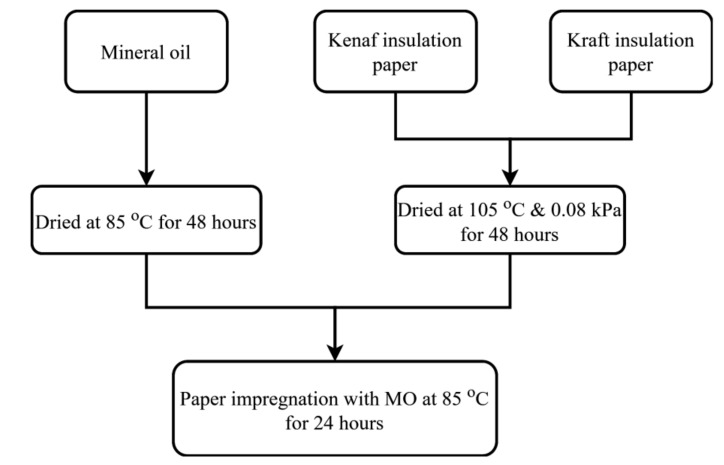
Pre-processing of oil and paper.

Kenaf papers obtained at different beating revolutions and different weight percentage concentration of PVA were examined for their physio-mechanical properties and AC breakdown voltage. The Kenaf papers were cut into the width and length dimensions of 15 mm and 150 mm, respectively. The Kenaf papers have thicknesses ranging from 92.99 μm to 175.90 μm with grammage of 52 g/m^2^. Kraft paper with thickness and width dimensions of 70 μm and 16 mm with a grammage of 52 g/m^2^ was also tested for comparison purpose. The Kraft paper was cut individually into the length of 150 mm. Mineral Oil (MO) was used to impregnate all of the papers. The pre-processing procedure of the oil and paper can be seen in Figure 8.

First, the MO was filtered three times by a membrane filter with a pore size of 0.2 µm. Next, the MO was dried in an air circulating oven at 85 °C for 48 h. The papers were dried in a vacuum oven at 105 °C at the pressure of 0.08 kPa for 48 h. The impregnations of both types of papers were carried out by the MO in an air circulation oven at 85 °C for 24 h.

### 3.2. AC Breakdown Voltage

**Figure 9:**
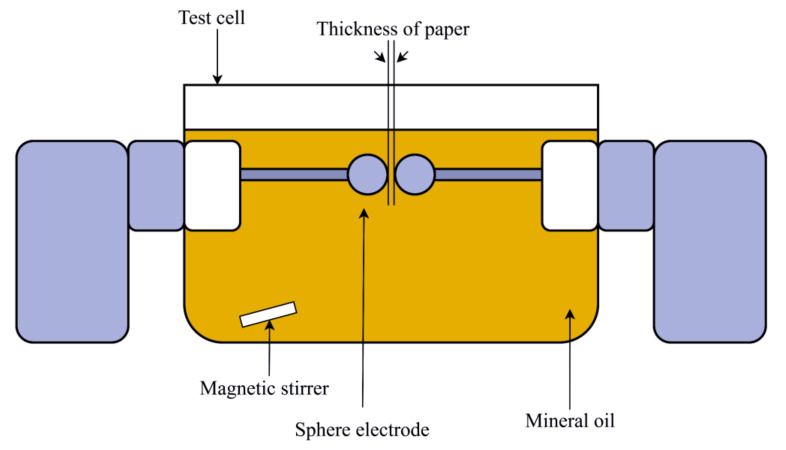
AC breakdown voltage test of paper.

The AC breakdown voltage was performed with BAUR DPA 75 C tester based on IEC 60156 [38]. Before the test was carried out, the test cell was drained, whereby the walls, electrodes, and test cells were rinsed three times with MO. In order to prevent the formation of bubbles, 400 mL of pre-processed MO was poured slowly into the test cell. The distance between the electrodes was adjusted according to the thickness of the paper sample. The AC breakdown test was carried out with two layers paper, since the measurement for one layer could not be computed since the thickness is too small. The test was carried out using spherical electrodes with diameter of 12.5 mm, with both sides facing each other. The voltage ramping rate during the test was 2 kV/s. The oil-impregnated paper was placed in between the electrodes, as shown in Figure 9. The paper sample was moved to other positions after each time of the breakdowns. A total of 20 AC breakdown voltages were recorded and the average value was used for analysis.

### 3.3. Scanning Electron Microscopy (SEM)

A Scanning Electron Microscope (SEM) was used to observe the arrangement of fibers and its bonds. The SEM imaging was carried out with COXEM EM-30ax. For the preparation of samples for SEM imaging, an ion coater COXEM SPT-20 was used to cover the surface of the paper sample with a thin conductive layer in order to avoid charging effect on the obtained image.

### 3.4. Apparent Density of Paper

**Figure 10:**
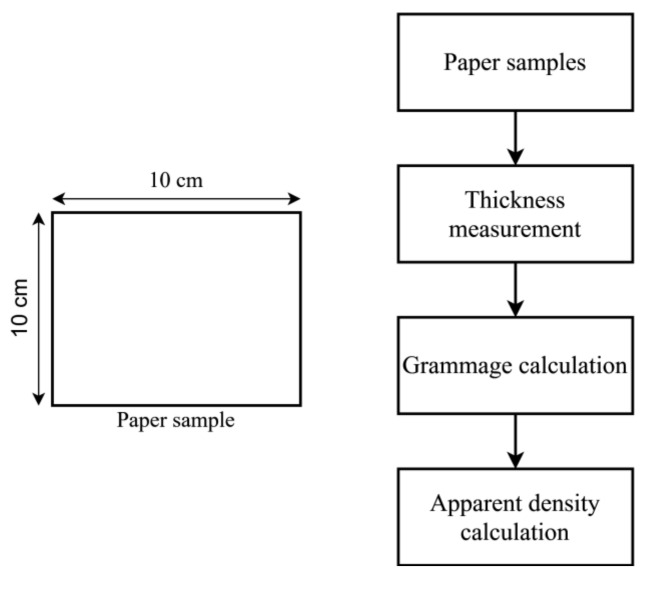
Grammage and apparent density measurements.

Ten paper samples were cut into the dimensions of 10 × 10 cm, as shown in Figure 10. The thickness of each paper sample was measured by L&W micrometer based on TAPPI T411 [39]. The sample’s grammage was determined based on Equation (4), by first measuring the sample’s mass and area, as per TAPPI T 410 [40]. The mass of each sample was measured using A&D analytical balance. The apparent density was calculated based on Equation (5).

(4) $$Grammage\;\left(g/m^{2}\right)=\frac{Mass\;\left(g\right)}{Area\;\left(m^{2}\right)}$$

(5) $$Apparent\;density\;\left(g/cm^{3}\right)=\frac{Grammage\;\left(g/m^{2}\right)}{Thickness\;\left(µm\right)}$$

### 3.5. Mechanical Properties of Paper

**Figure 11:**
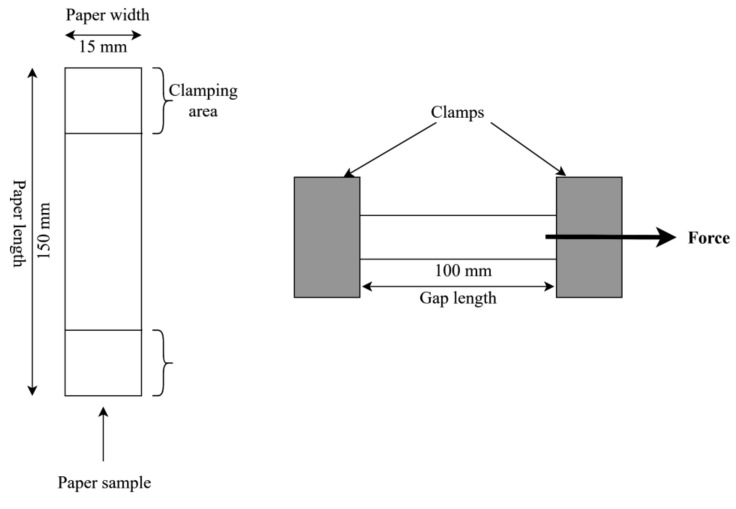
Tensile strength measurement.

A Buchel B.V horizontal tensile tester was used for measuring the paper tensile strength, as per TAPPI T494 [41]. The measuring gap length of the test was set to 100 mm ± 1 mm. The length and width of the paper sample were 150 mm and 15 mm respectively, as shown in Figure 11. The crosshead speed was set to 20 mm/min. [41]. The test was carried out at 23 °C ± 1 °C and 50% ± 2% relative humidity. The TI was calculated based on Equation (6).

(6) $$TI\;\left(Nm/g\right)=\;\frac{Tensile\;strength\;\left(N/m\right)}{Grammage\;\left(g/m^{2}\right)}$$

**Figure 12:**
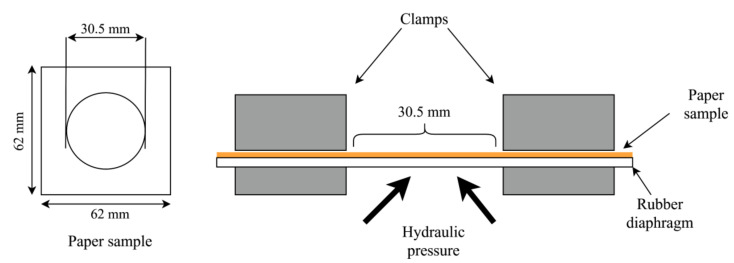
Burst strength measurement.

The burst strength is the maximum hydrostatic pressure that is required to produce rupture of the paper when a controlled and constantly increasing pressure is applied through a rubber diaphragm to a circular area of 30.5 mm, as seen in Figure 12. The burst strength of paper was measured by Frank burst machine based on TAPPI T 403 [42]. A total of 10 paper samples with dimension of 62 mm × 62 mm were prepared. The test was carried out at 23 °C ± 1 °C and 50% ± 2% relative humidity. The paper sample was placed in between the clamps with a clamping pressure of not more than 1200 kPa [42]. A constant increasing hydraulic pressure was applied to the paper through a rubber diaphragm, until the paper bursts. The BI was determined based on Equation (7).

(7) $$BI\;\left(kPa.m^{2}/g\right)=\frac{Burst\;Strength\;\left(kPa\right)}{Grammage\;\left(g/m^{2}\right)}$$

**Figure 13:**
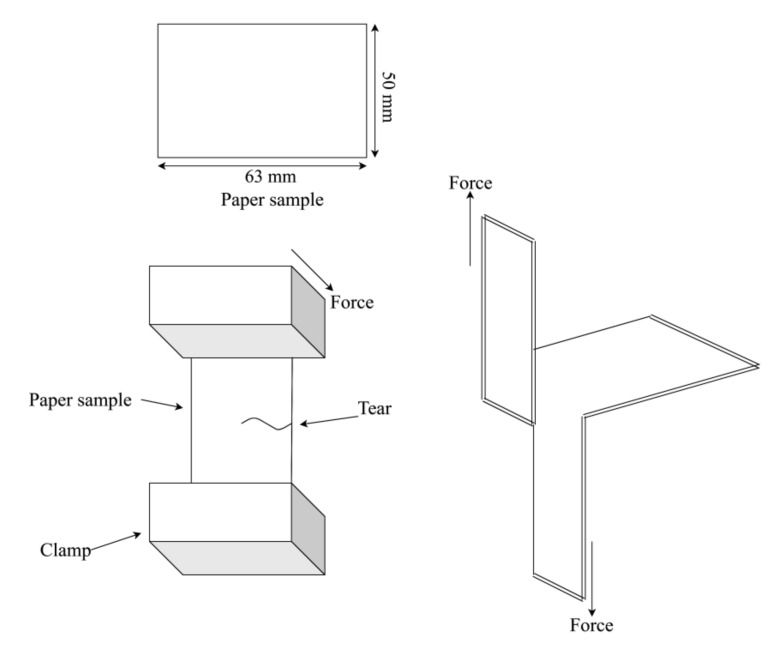
Tear strength measurement procedure.

The tear strength is the force that is required to tear the paper. The tear strength of the paper was measured by Elmendorf tearing tester, as per TAPPI T414 [43]. Ten samples with the dimensions of 63 mm × 50 mm were prepared. The test was carried out at 23 °C ± 1 °C and 50% ± 2% relative humidity. The paper sample was placed in between the clamps with the same clamping pressure of 0.55 MPa on both sides, as seen in Figure 13. A slight cut in the middle of the paper was made before the force was applied [43]. The tear strength was recorded and the TeI was determined based on Equation (8).

(8) $$TeI\;\left(mN.m^{2}/g\right)=\frac{Tear\;Strength\;\left(mN\right)\;}{Grammage\;\left(g/m^{2}\right)\;}$$

## 4. Results and Discussion

### 4.1. Kenaf Pulp Properties

Table 1 illustrates the characteristics of Kenaf fibers. The standard Kenaf bast fibers are typically up to 3 mm long with width between 13.8 μm and 19.5 μm [10,18]. According to [10,18], the lumen width of the Kenaf bast fibers is between 4.3 μm and 10.1 μm, while the average cell wall thickness is 11.90 µm [44]. All the properties of the tested Kenaf fibers are within the ranges, as in [10,18], except for the cell wall thickness, which is slightly low.

Without beating, the freeness of Kenaf pulp is 718 mL [45]. It is quite high, and it indicates that the paper is porous to liquids. As a result, the paper can lose its strength if impregnation is carried with MO. The standard range of pulp freeness for a strong paper with good mechanical strength is between 100 mL and 200 mL [45]. The strength of the paper is one of the main requirements for transformers application. Beating could reduce the freeness of the pulp, which eventually increases the paper’s mechanical strength [33,45]. The beating of 12,000 revolutions could decrease the freeness of the pulp up to 92%, as the beating of the pulps helps to break the fibers into more fibrous fragments [45].

### 4.2. Morphological Analysis of Kenaf Fiber

**Figure 14:**
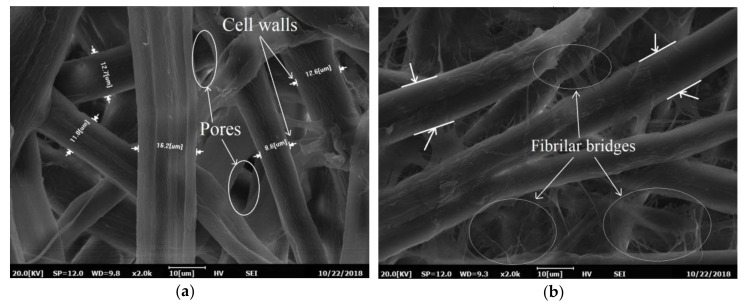
Scanning electron microscope (SEM) images of Kenaf paper at magnification of ×2000 (**a**) unbeaten pulp (**b**) beaten at 12,000 revolutions.

Figure 14a illustrates the SEM images of the unbeaten Kenaf paper taken at magnification of ×2000. The fiber width is between 8.6 µm and 16.2 µm with an average of 12.4 µm that is close to the measurement by a fiber classifier, as shown in Table 1. The fiber walls are free from fibrils that suggest that there is no fibrillation on fiber walls and signify a clean network of fibers as shown in Figure 14a. Because the fiber wall for unbeaten pulp is not fibrillated, the pores are quite apparent and could promote the flow of oils. After beating, the fibrillation starts to occur as shown in Figure 14b. There are a number of fibrilar bridges on the fiber cell walls, which promote new bonding and subsequently increase the strength of the Kenaf paper through an increment of the contact area and density. The structures of the fiber walls surface and the cell walls of unbeaten pulp are quite smooth when compared to beaten pulps, as shown in Figure 14.

### 4.3. Effect of Mechanical and Chemical Treatments on the Structure of Kenaf Fibers

**Figure 15:**
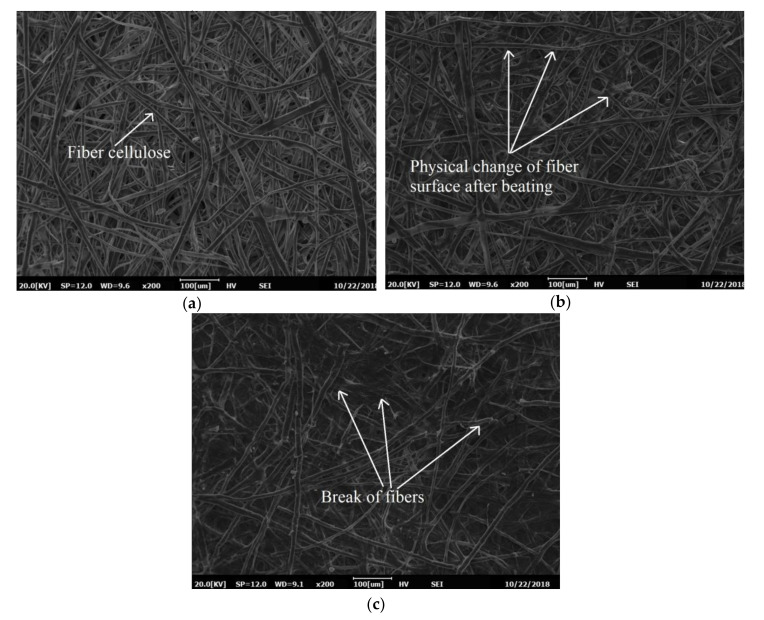
SEM images of Kenaf paper: (**a**) unbeaten pulp; (**b**) beaten at 6000 revolutions; and, (**c**) beaten at 12,000 revolutions.

Figure 15 shows the SEM images of the Kenaf paper under mechanical treatments. Figure 15b illustrates that the beating of 6000 revolutions improves the number of fibrils, which lead to the increment of the fiber joints. The number of fibrils is further enhanced after 12,000 revolutions of beating with the increment of the fiber flexibility, as seen in Figure 15c. The new fibrils are bonded to each other to increase the contact area. The increment of the contact area results in the increment of apparent density of paper. The beating process affects fiber surfaces, as there are many fibrils on cell walls, leaving behind uneven surfaces on the fiber cell wall. These fibrils bond to form the fibrilar bridges, resulting in an increment of the fibril network. As the fibrils bonding increases, the BI of the paper increases. The beating process results in fibrillation on the fiber cell walls and lead to the improvement of the paper tensile strength, as shown in Figure 15c [45].

The physio-mechanical properties of the Kenaf paper at different beating revolutions have been previously examined in [45]. It is found that the beating at 12,000 revolutions could provide the highest improvements of the TI and BI as well as density. On the other hand, the TeI and thickness decrease. The beating process increases the flexibility of the fiber, bonding, and lead to denser fiber. As a result, high tensile forces and pressures are required to break the Kenaf fibers. On the other hand, the beating process results in shorter fibers whereby less force is required to tear the paper and, hence, lower TeI. Because the Kenaf paper beaten at 12,000 revolutions results in the highest performance, it has been chosen for further improvement through introduction of PVA.

**Figure 16:**
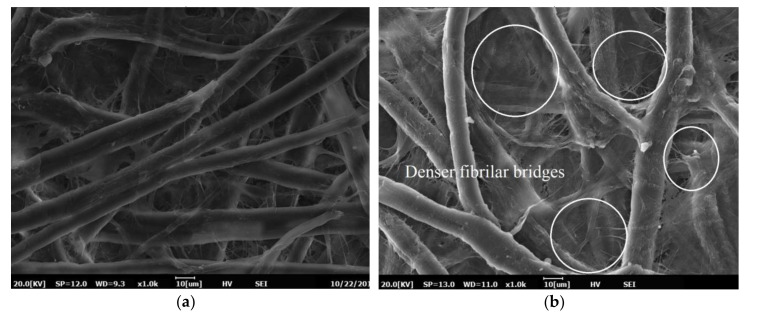
SEM images of Kenaf paper: (**a**) beaten at 12,000 revolutions; and, (**b**) beaten at 12,000 revolutions and 12% PVA.

Figure 16 illustrates the SEM images of Kenaf paper after chemical treatment. The introduction of the PVA to the pulp promotes fibrillation bridges through the presence of several cellulose strands and subsequently enhances the network bonding of the fibers. Apparent fibrils are bonded on the fiber cell walls, as seen in Figure 16a,b. This network increases the number of bonds and reduces the number of the pores in the Kenaf paper.

### 4.4. Physio-Mechnical Properties of Kenaf Paper with PVA

**Figure 17:**
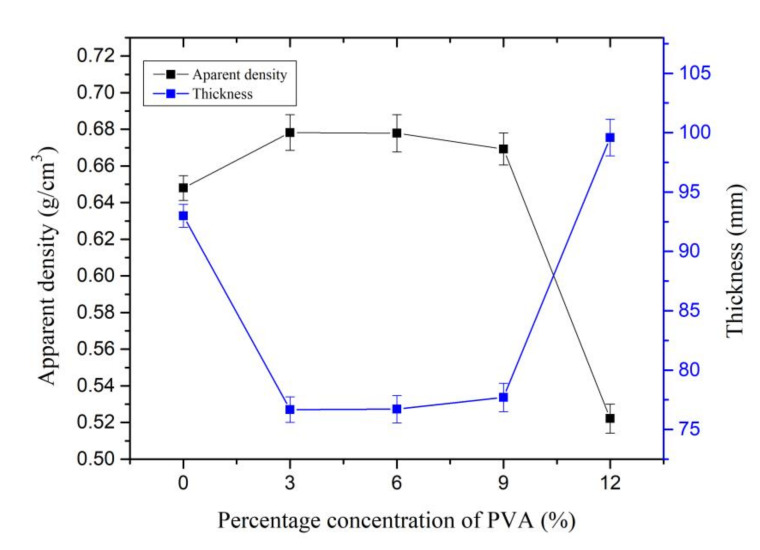
Thickness and density at different weight percentage concentration of PVA.

The Kenaf paper thickness decreases with the addition of 3% of PVA and it increases as the PVA increases from 9% to 12% of PVA, as shown in Figure 17. The thickness of the paper increases by 7.09% as 12% of PVA is introduced. Initially, the density of the Kenaf paper slightly increases as 3% of PVA is introduced. The density of the Kenaf paper maintains almost unchanged as the PVA increases from 3% to 9%. The density of the Kenaf paper significantly decreases by 19.44% as 12% of PVA is introduced.

### 4.5. Mechanical Properties of Kenaf Paper with PVA

**Figure 18:**
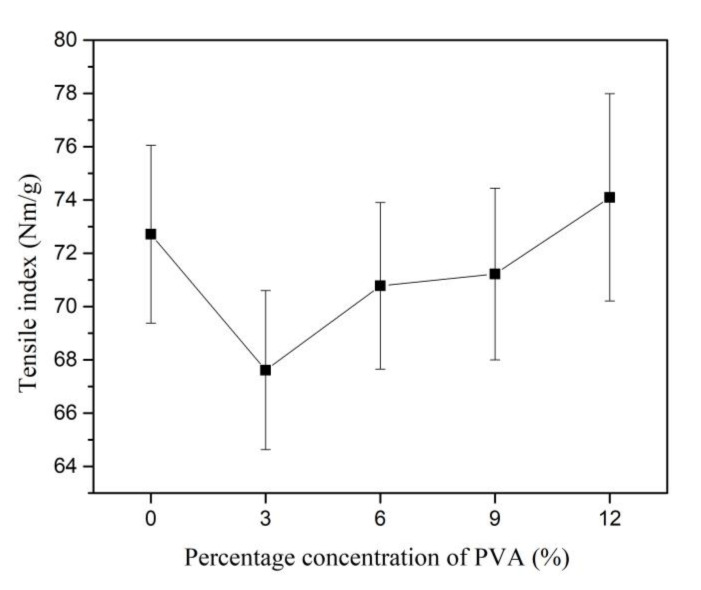
Tensile index of Kenaf paper at different weight percentage concentration of PVA.

The TI of Kenaf paper initially decreases by 7.01% as 3% of PVA is introduced. As the weight percentage concentration of PVA increases from 3% to 12%, the TI of Kenaf paper increases, as shown in Figure 18. Based on SEM imaging in Figure 18, fibrillation occurs as PVA is introduced and leads to the increment of hydrogen bonding, which subsequently increases the strength of the Kenaf paper. As compared to without PVA, the highest increment of TI of paper is 1.89% at 12% of PVA. The coefficient of variation is between 1.8% and 4.3% as the weight percentage concentration of PVA increases to 12%. However, the TI of Kenaf paper is still considered low when compared to Kraft paper with the strength of 113 Nm/g.

**Figure 19:**
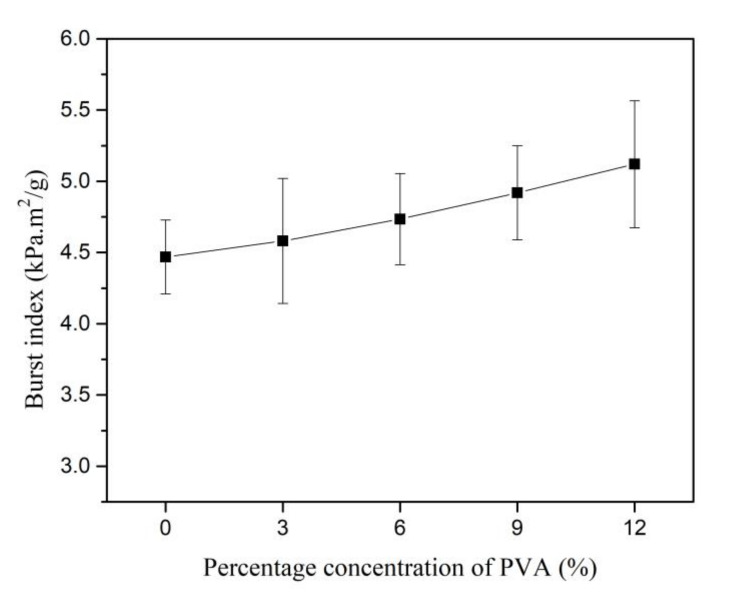
Burst index at different weight percentage concentration of PVA.

The BI of Kenaf paper increases steadily with the increment of weight percentage concentration of PVA, as shown in Figure 19. The introduction of PVA in the paper tends to increase the fibrilar bridges and increases hydrogen bonding. Consequently, higher pressure is required to break the bonding between fibers. The BI of Kenaf paper increases by 14.54% as 12% of PVA is introduced. The coefficient of variation fluctuates between 24.5% and 15.7% as the weight percentage concentration of PVA is increased to 12%.

**Figure 20:**
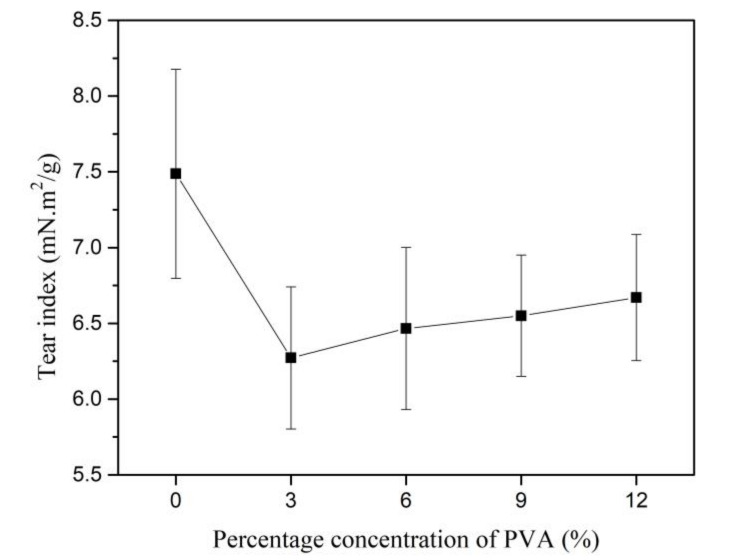
Tear index at different weight percentage concentration of PVA.

The pattern of the TeI is similar to the TI of Kenaf paper, as shown in Figure 20. The TeI initially decreases as the weight percentage concentration of PVA is increased to 3%. The TeI slightly increases at much lower rate than TI as the weight percentage concentration of PVA increases from 3% to 12%. The range for the coefficient of variation is between 1.5% and 7.5% as the weight concentration of PVA is increased up to 12%. TeI is related to the length of fibers. The addition of PVA to the pulp does not further increase the fiber length; therefore, it is expected that TeI would not be increased as the weight percentage concentration of PVA increases.

### 4.6. AC Breakdown Voltage of Kenaf Paper without PVA

**Figure 21:**
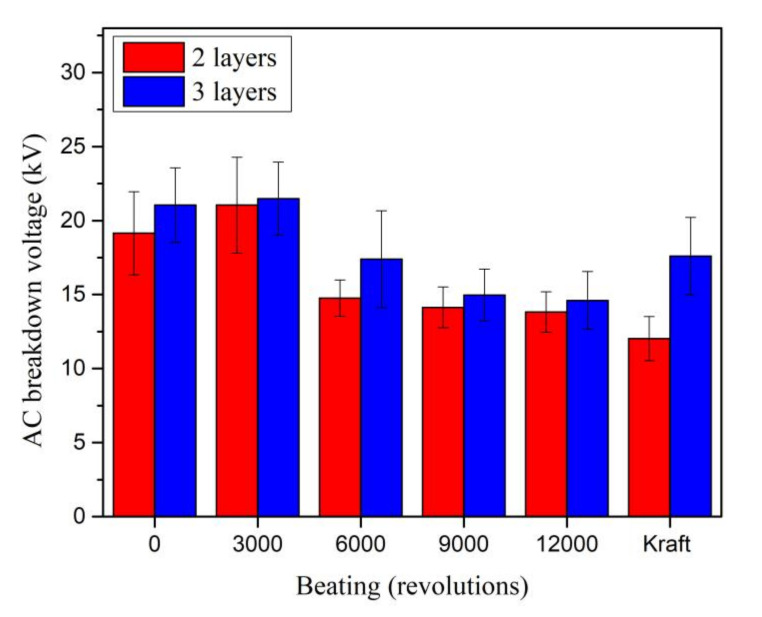
AC breakdown voltage of Kenaf and Kraft papers at different layers and beating revolutions.

**Figure 22:**
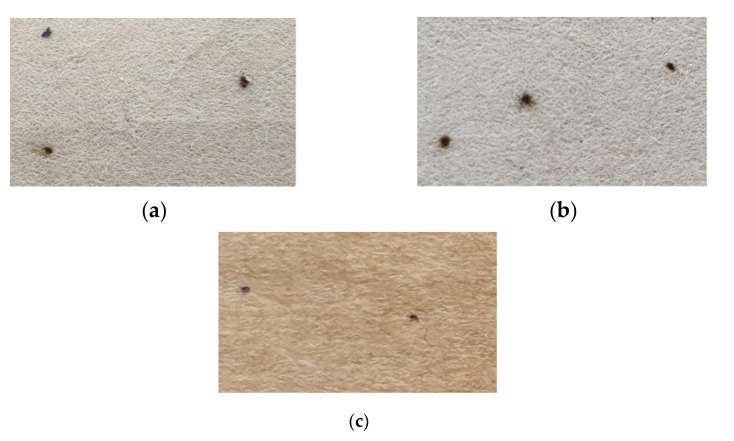
Paper condition after breakdown tests: (**a**) Unbeaten Kenaf paper; (**b**) Kenaf paper with 12,000 revolutions of beating; and, (**c**) Kraft paper.

The AC breakdown voltages of the MO impregnated Kenaf papers for two and three layers are observed, as seen in Figure 21. The pattern of the Kenaf and Kraft paper still comply with the existing multiple layer effect whereby the AC breakdown voltage increases with the increment of paper layers [46,47,48,49]. The AC breakdown voltage patterns of Kenaf paper for both layers are quite similar as the beating revolution increases. With the increment of beating revolution to 3000, the AC breakdown voltages of Kenaf paper for two and three layers slightly increase to 9.8% and 2%, respectively. As the beating revolution increases to 6000, the AC breakdown voltages of Kenaf paper for two and three layers decrease to 22.8% and 17.3%, respectively. At beating of 12,000 revolutions, the AC breakdown voltage of Kenaf paper for two layers is 14.92% higher than Kraft paper. Meanwhile, the AC breakdown voltage Kraft paper for three layers is 20.49% higher than the Kenaf paper. The paper condition after the AC breakdown tests can be seen in Figure 22.

**Figure 23:**
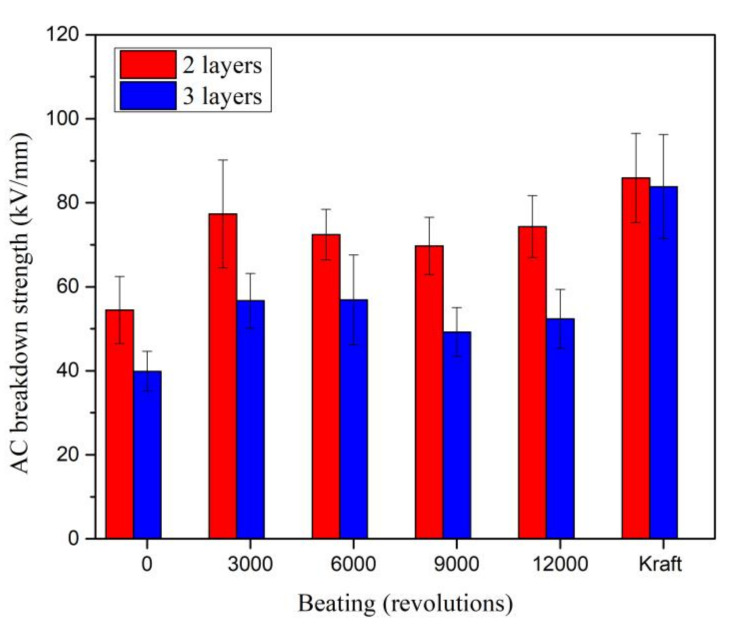
AC breakdown strength of Kenaf and Kraft papers at different layers and beating revolutions.

The AC breakdown strength was obtained by dividing the AC breakdown voltage with the thickness of paper, since it was set as the gap distance. Similar as AC breakdown voltage, the AC breakdown strengths of Kenaf paper for two and three layers slightly increase to 42.05 % and 42.12 % as the beating increases to 3000 revolutions as shown in Figure 23. The AC breakdown strengths of Kenaf paper for two and three layers fluctuate between 72.4 kV/mm and 74.3 kV/mm as the beating revolution increases from 6000 to 12,000. At the beating of 12,000 revolutions, the AC breakdown strengths of Kenaf paper for two and three layers are 13.49 % and 37.52 % lower than the Kraft paper.

### 4.7. AC Breakdown Voltage of Kenaf Paper with PVA

**Figure 24:**
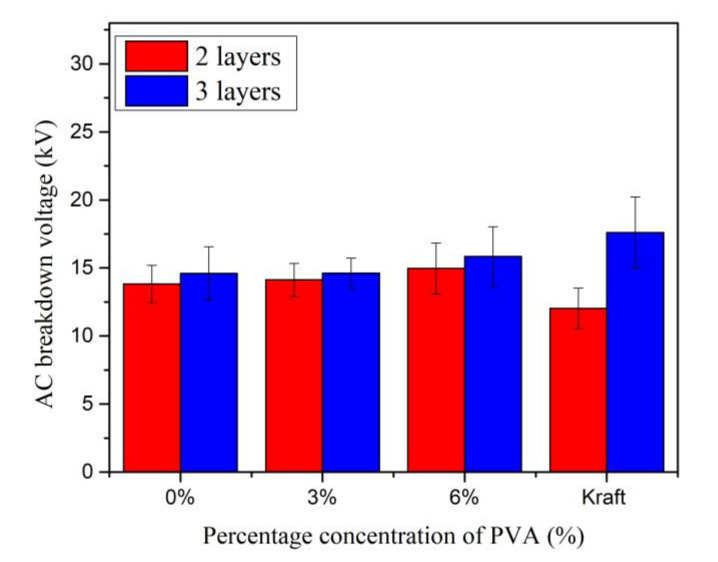
AC Breakdown voltage of Kenaf and Kraft papers at different weight percentage concentration of PVA.

**Figure 25:**
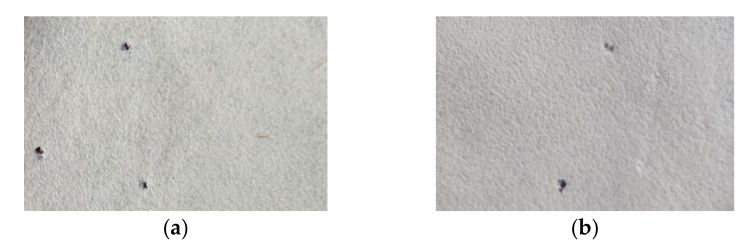
Paper condition after breakdown tests: (**a**) Kenaf paper with weight percentage concentration of 3% of PVA and (**b**) Kenaf paper with weight percentage concentration of 6% of PVA.

The AC breakdown voltage of MO impregnated Kenaf paper with PVA weight percentage concentrations of 3% and 6% can be seen in Figure 24. The AC breakdown voltages of Kenaf paper for two and three layers increase almost linearly with the increment of the weight percentage concentration of PVA. At PVA weight percentage concentration of 6%, the AC breakdown voltages of Kenaf paper for 2 and 3 layers increase by 8.2% and 8.4%. The AC breakdown voltage of Kenaf paper for 2 layers at PVA weight percentage concentration of 6% is 24.36% higher than Kraft paper. On other hand, the AC breakdown voltage of Kenaf paper for three layers is 10.03% lower than Kraft paper at the same weight percentage concentration of PVA. Figure 25 illustrates the paper condition after the breakdown tests.

**Figure 26:**
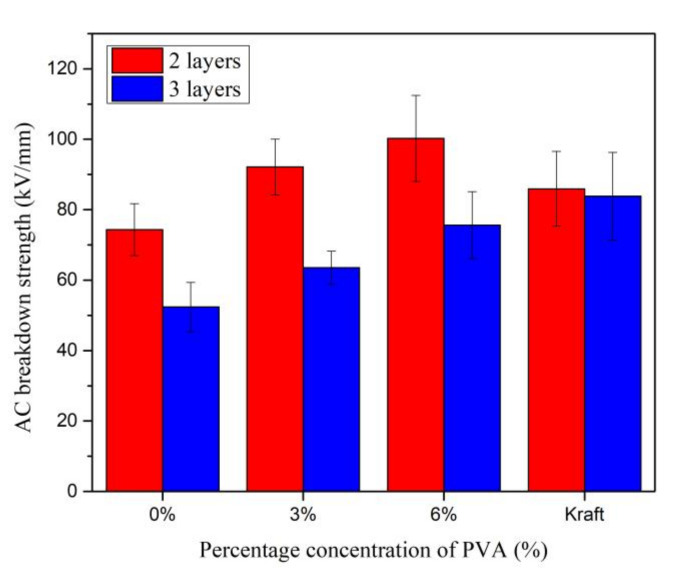
AC Breakdown strength of Kenaf and Kraft papers at different weight percentage concentration of PVA.

The AC breakdown strengths increment patterns of Kenaf paper for two and three layers are quite apparent, as shown in Figure 26. As the PVA weight percentage concentration increases to 6%, the AC breakdown strengths of Kenaf paper for two and three layers increase by 34.88% and 44.37%. Similar to the AC breakdown voltage, the AC breakdown strength of Kenaf paper for two layers is 16.68% higher than Kraft paper at the same PVA weight percentage concentration. The AC breakdown strength of Kraft paper is 10.87% higher than the AC breakdown strength of Kenaf paper for three layers.

## 5. Discussion

The mechanical properties of the paper arise from the interfiber bonding which could occur between two fibers through either the van der Waals interaction or molecular linkage [50]. Fiber bonding could affect the optical, electrical, and structural properties of the paper [50,51,52].

Interfiber bonding occurs once the water is extracted during the papermaking process, whereby the surface tension forces pulling fibers together [53]. Through the drying process, the pulp fibers could laterally shrink, which cause shear stress to the bonding area. It is due to the differences between the tendency of lateral and longitudinal shrinkage of fibers [54]. The shrinkage is dependent upon the revolutions to which the wet fiber wall swells, and it is influenced by internal fibrillation as well as the composition of the chemical properties of the fiber wall. Shrinking forces are the strongest in the peripheral area of bond and the bonding edges bear the load first when loaded. Shrinkage stress induces axial compression force on the crossed fibers, and it can cause deformations in the segments of bonded fibers [55]. Stresses at the bonding area and fiber wall change the mechanical characteristics of the bonded fibers and subsequently affect the strength of the paper [56]. Activation is one of the related fiber characteristics of a network [57,58]. The kinky, curly, or deformed fiber segments, which cannot be loaded to the networks, could originally be changed into active network components [55,56,57]. The process of activation occurs during the drying process of papermaking. Fiber morphology could affect the required drying stress to enable the free segments. Activating the free segments not only makes the segment straighter and more capable of holding load, but it can also raise the order of cellulose and hemicelluloses within the fibrils and decrease the fibril angle.

Flexible fibers may form bonds, fibrils, and fines from bridges between fibers. Through the increment of the fibers contact, the fibers surface physically changed, which affect the physical properties of paper. Delamination, swelling, and dislocation among the individual fibers could be performed through beating, which results in increment of fibers flexibility [57]. In Figure 14b, it is apparent that the large number of fibrils on the cell walls creates new bonds and increases the area of contact.

Bonding between fibers consists of the strength of a single fiber and interfiber bonds [57,58]. The TI and BI depend on the strength of interfiber bonds, while TeI depends on the strength of single fiber [56,57,58,59]. It is evident from the analysis that the TI and BI increase with the increment of the beating revolution, as in [45]. It is because the beating process enhances the interfiber bonding through increment of the fiber flexibility. On the other hand, the TeI, which is a function of strength of single fiber, decreases with the increase in beating revolution. Beating results in shorter fiber length, which causes a decrement of the tear strength.

It is shown that PVA could affect the physio-mechanical performances of the Kenaf paper, as shown in Table 2. It is shown that the apparent density decreases by 19.44% as 12% of PVA is introduced. The thickness, TI, and BI increase by 7.09%, 1.89%, and 14.54% with 12% addition of PVA. The introduction of PVA to the pulp induces new molecular linkage with the fiber cell walls, which increases the fibrilar bridges development on the fiber cell walls, as seen in Figure 16b. As TI and BI parameters are directly proportional to interfiber bonding, therefore the increment of the fibrilar bridges suggest that bonding in between fibers increases, resulting in an improvement of the mechanical strength of the paper. However, PVA does not affect the tear strength of the paper as the tear strength of the paper is related to the strength of the fiber itself. It is interesting to observe the AC breakdown voltage and strength of the Kenaf paper decrease with the increment of the beating revolution. It is anticipated that the decrement of the pore volumes due to the beating leads to the high probability of the breakdown to occur in the Kenaf fibers instead mainly through the discharge channels in pores that are filled with the MO [60]. On other hand, the AC breakdown voltages of Kenaf paper for two and three layers slightly increase as the weight percentage concentration of PVA increases from 3% to 6%. It is known that the electrical and physiochemical/mechanical strength of solid insulation could be improved through the introduction of enhancement materials [60,61]. In the current study, PVA is found to be one of the enhancement substances that can be used to further improve the AC breakdown properties of the Kenaf paper. Nevertheless, further study is required to seek further understanding on the detail mechanisms of breakdown for Kenaf paper under beating and PVA enhancement.

## 6. Conclusions

The beating process increases the flexibility of the fiber, bonding and leads to denser fiber for the Kenaf paper. Subsequently, this process will lead to the improvement of the physio-mechanical properties. With the introduction of PVA, the TI and BI could increase by 1.89% and 14.54%, while the TeI decreases by 10.94%. On the other hand, the thickness increases to 7.09% and density decreases to 19.44% with the introduction of PVA. The AC breakdown voltage for multiple layers of MO impregnated Kenaf paper decreases as the beating increases. On the other hand, the AC breakdown voltage of MO impregnated Kenaf paper for two and three layers increases as the weight percentage concentration of PVA increases. Further analysis shows that the AC breakdown strength of Kenaf paper is comparable with Kraft paper. The TI of Kenaf insulation paper with PVA is 1.89% higher than without PVA, but 34.42% lower than the Kraft paper. It is concluded that PVA could further improve a few of the physio-mechanical and electrical properties of Kenaf paper. However, other enhancement materials could be introduced to Kenaf paper in order to ensure its viability as one of the insulation materials in transformers.

## Figures and Tables

**Table 1:** Fiber characteristics.

Property	Value
Fiber length (mm)	2.964 ± 0.482
Fiber width (µm)	12.24 ± 2.84
Lumen width (µm)	8.08 ± 2.12
Cell wall thickness (µm)	2.08 ± 0.7

**Table 2:** Physio-mechanical properties of Kenaf paper without and with PVA.

Property	Without PVA	With 12% of PVA
Apparent density (g/cm^3^)	0.648	0.522
Thickness (µm)	92.99	99.59
Tensile index (Nm/g)	72.72	74.1
Burst index (kPa.m^2^/g)	4.47	5.12
Tear index (mN.m^2^/g)	7.49	6.67
